# Recovery Strategies in Elite-Level Male Rugby Union Players and Positional Considerations: A Scoping Review

**DOI:** 10.1186/s40798-026-01056-3

**Published:** 2026-07-01

**Authors:** Adam Grainger, Robert Allan, Giampiero Tarantino

**Affiliations:** 1 University College Dublin, Dublin, Ireland; 2 Ulster Rugby, Belfast, Northern Ireland; 3 School of Health, Social Work and Sport, University of Lancashire, Preston, England; 4 University of Southern Denmark, Odense, Denmark

**Keywords:** Forwards, Backs, Fatigue, Muscle damage, Recovery modalities

## Abstract

Rugby union training and competition results in task-specific fatigue. As a result, practitioners working in elite rugby settings must identify and understand the position-specific demands placed upon players. Aligning appropriate recovery strategies is essential to optimise performance. Therefore, the primary aim of this scoping review is to explore current fatigue and recovery strategies in elite rugby players and investigate any specific strategies used to enhance recovery. The secondary aim of this scoping review is to discuss potential positional differences in relation to recovery strategies and fatigue markers. Thirty-seven articles were included in this scoping review, with results identifying a diversity in the recovery approaches taken, with cold water immersion the most commonly used, compression garments, partial and whole-body cryotherapy, electrostimulation, nutritional supplements (fish oil), pool-based active recovery, and innovative mattresses also utilised. The findings align with previous research showing that many recovery strategies have limited empirical support; however, an interesting finding from our review was that 26 studies included no specific recovery intervention (natural recovery) within their research methodology. Only five studies reported positional data results, meaning it is difficult to appropriately compare position-specific recovery or quantify the effectiveness of specific recovery strategies. Thus, this scoping review serves as a “call to arms” to the rugby union research community to identify position-specific data in future studies and then integrate positional specificity and individualised athlete needs to enhance recovery. Given the limited evidence that this scoping review has provided, there is a need to further explore the position-specific recovery strategies in future studies to assess their potential impact on performance.

## Key Points

- Fatigue post rugby union match play is commonplace and position specific
- Recovery modalities used to hasten recovery time course in elite rugby union vary, with limited empirical evidence existing for many of such modalities
- Cold water immersion was the most commonly used recovery modality, with compression garments, partial and whole-body cryotherapy, electrostimulation, nutritional supplements (fish oil), pool-based active recovery, and innovative mattresses also utilised
- A need exists to further explore the position-specific recovery strategies in future studies to assess their potential positive impact on recovery time course and subsequent performance

## Introduction

Elite-level rugby union is played professionally and internationally and involves intermittent high-speed collisions (> 59, above 13G), rapid decelerations (> 50, > 3 m/s^2^) [1], and high running volumes (5850 m forwards, 6545 m backs) [1–5]. Regular rugby training and competition can compromise physiological function, directly impacting sporting performance and/or reducing the capacity to regularly train at the desired intensity [6, 7]; thus, symptoms of post-match fatigue are known to remain present for multiple days [8] and accumulate across a competitive season [9].

Elite rugby union players are required to return to training in the immediate days post-match to prepare for the next [8, 10]. As a result, players often experience having to play and train amidst symptoms of fatigue throughout subsequent weekly microcycles [11]. This creates an inherent tension between prescribing adequate training load to maintain fitness while ensuring sufficient recovery to optimise readiness for the next match within a congested weekly microcycle. Fatigue is known to be transient and, when well-managed, typically dissipates before the next competition or training session [12]. However, if fatigue is not appropriately controlled, further training can have the potential to delay or exacerbate performance and/or perceptual decrements post-match play. For the modern rugby union player, it is essential that recovery can be achieved quickly and optimally so that the required levels of performance can be maintained (or improved) across multiple sessions and/or competitions [13]. Recovery is defined as the ability to return the body to a pre-exercise state, as such, where sufficient time is not available for this to occur naturally, i.e. without a specific modality intervention, any imbalance between the stress of training and competition, and recovery may have the potential to lead to not only performance decrement but also non-functional overreaching [13, 14]. Alongside the definition of recovery, throughout this scoping review, we define the term “fatigue” to describe measurable decrements in physiological capacity, neuromuscular function, biochemical markers, endocrine balance, and/or subjective well-being experienced by players following match play or training. This operational definition is important because fatigue, in its strict sense, implies a functional decrement or perturbation in one or more of these domains. Consequently, the term “fatigue” is not universally applicable to all players across all conditions. For example, players who do not complete full match exposure, play in specific positions with lower absolute loads, or who demonstrate resilience to the physiological demands of match play may not experience measurable fatigue across all assessed markers. In such cases, alternative terminology, such as the influence of match play and/or training, post-match response, or post-match perturbations, may be more technically precise.

Fatigue experienced by rugby players after match play or training is complex and task specific and can be attributable to many factors, all of which will be dependent on the demands imposed upon the player during the activity. For example, physical loads such as distance covered, the number and intensity of collisions (while contesting possession in attacking or defensive situations), sprinting (including acceleration), jumping, decelerations, and changes of direction [15] will all influence factors modulating performance and perceived fatigability. The goal of many practitioners working within rugby union is therefore to account for such fatigue and plan to restore players' state of readiness back to pre-match levels in the shortest possible time. The ability to identify and understand the specific demands placed upon rugby players during match play and training situations has therefore been recognised as a crucial factor in developing appropriate training and recovery programmes which may elicit improved performance [1, 4, 16–18].

Forwards typically perform high-intensity static exertion (e.g. scrums, rucks, mauls) for longer periods than backs [18, 19], with absolute and relative demands also differing between forwards and backs [4, 20]. Mashiko and colleagues [21] reported that rugby union backs display movement patterns more focused on high-speed running and tackling, compared to rugby union forwards, who generally take part in running, tackling, and an element of mauling and scrummaging involving physical contact. Blunt trauma associated with forward play may result in more prolonged muscle damage than eccentric actions, more typical of backs, due to their greater number of decelerations and accelerations completed within game situations compared to forwards. Further data from rugby union [22] support the view that the number of impacts encountered during a match relates specifically to the levels of muscle damage (high creatine kinase [CK] levels) experienced. When considering that forwards play less time and cover less distance than backs, yet experience longer muscle damage [23], the detrimental effect of the blunt force trauma that forwards experience is further emphasised.

Coughlan et al. [16] reported that players completed 75% of their match activities at low intensity, with backs entering high-intensity zones more frequently, while the forward was exposed to a higher number of impacts and total body load measured via an accelerometer. Despite forwards completing the majority of their activities at a low-speed intensity, their heart rate is still within high-intensity ranges. During periods of low-speed intensity, forwards could be recovering from high-intensity bouts of activity, or involved in static movements such as rucks and mauls, which, despite being conducted at low speeds, involve high exertion [18, 19]. Backs, in contrast, were reported by Quarrie et al. [17] to move greater distances at speeds > 6 ms^−1^ compared to forwards. This greater distance at speeds > 6 ms^−1^ (252 m for forwards and 450 m for backs) was due to backs covering a greater average sprint distance than forwards when conducting their movement demands during games. Additionally, Jones et al. [22] reported that high-speed running was a predictor of muscle damage for backs, with tailored individual recovery strategies, based upon impacts and high-speed running data derived from GPS, being of interest.

Thus, differences in distances, intensities of effort, range of sprints, jumps, and contacts (with both opposition players and the playing surface) between positions inevitably create varied fatigue profiles that need to be managed appropriately by multidisciplinary practitioners supporting players between match and training exposures to optimally prepare players to perform to their potential. The idea of an individualised and/or tailored recovery programme is not a new idea, with recent discussion around appropriate recovery being individualised and periodised on an athlete-by-athlete basis [8, 13, 14, 24].Considering that fatigue may be position-specific and multifactorial (involving neuromuscular, biochemical, endocrine, and perceptual components), recovery approaches should be tailored to address these distinct stressors. However, despite these theoretical foundations, the current body of evidence examining position-specific recovery strategies in rugby union remains limited. Therefore, the primary aim of this scoping review is to explore fatigue and recovery in elite rugby players and investigate any specific strategy used to enhance recovery. The secondary aim of this scoping review is to discuss potential positional differences in relation to recovery strategies and fatigue markers. For the purposes of this research, natural recovery will also be considered as a recovery strategy. Within the context of this review, natural recovery is defined as a designated period of time when no recovery interventions were implemented to hasten recovery time course. Specifically, instead of using tools, methods, and/or strategies, during this natural recovery process, only common daily needs such as sleep, nutrition, and hydration were used [25]. Despite the use of natural recovery not involving specific intervention beyond the norm, this natural process and the data associated were of interest for the purpose of this scoping review to enable potential comparison within and between recovery strategy groups.

## Methods

This systematic scoping review was conducted following the Joanna Briggs Institute guidelines for scoping review [26] and the Preferred Reporting Items for Systematic Reviews and Meta-Analyses Extension for Scoping Reviews (PRISMA-ScR) was used for reporting this review [27]. The protocol for this systematic scoping review was registered in the Open Science Framework on the 17th of January 2025, and it is available at https://osf.io/vrfhm/?view_only=75aab83888c04d5eafc207b9fde69ab0 [28].

### Selection of the Source of Evidence and Search Strategy

Six electronic databases (PubMed, EMBASE, SportDISCUS, CINAHL, Web of Science, and Scopus) were searched up to January 2025, with four main concepts: (1) Rugby Union; (2) Professional Players; (3) Recovery; and (4) Fatigue. The primary search strategy was developed in PubMed, and it was then translated to match the features of the other databases. MeSH terms (PubMed), thesaurus (Emtree in EMBASE and SportDISCUS), and subject headings (CINAHL) were also used. The full search string used in PubMed was the following: ((((Rugby union) OR ("Rugby"[Mesh])) AND ((((Player*) OR (athlet*)) OR (professional*)) OR ("Athletes"[Mesh]))) AND (((recover*) OR (rest*)) OR ("Rest"[Mesh]))) AND ((((Fatigue) OR (Demand*)) OR ("Fatigue"[Mesh])) OR ("Muscle Fatigue"[Mesh])). The full search strings for each database are provided in the protocol material. We applied no date restrictions and limited the search to papers written in English.

### Eligibility Criteria

Inclusion criteria for this scoping review comprised articles that (a) investigated male rugby union professional players; (b) used any type of recovery strategies (for example, cold water immersion, or natural recovery); (c) assessed fatigue and/or performance (e.g. neuromuscular, biochemical); (d) reported match or rugby-specific activity; and (e) employed any type of research design (both quantitative and qualitative design). The exclusion criteria that informed the screening process were peer reviewed studies that (a) explored rugby league and rugby sevens; (b) had a population of non-professional players (amateur, youth, semi-professional, etc.); (c) had a population of female players; (d) had a population of under 18 years old; (e) were conference proceedings; and (f) exposed the players to non-rugby-specific movement (i.e. Yo-Yo test). Considering the exploratory nature of the research questions and the absence of inferential statistics, a critical appraisal was deemed not applicable to this systematic scoping review. Reviews were excluded, but the references included in the reviews were screened to find potentially relevant articles.

### Screening Process

After articles were retrieved from the electronic databases, three reviewers (authors' initials anonymised for peer-review) independently carried out the title/abstract screening process in Covidence (Veritas Health Innovation). Any discrepancies at this stage were discussed between the three reviewers until an agreement was reached. The full texts of the potential suitable articles were then screened by two authors, with a proportionate agreement of 0.76, and Cohen’s Kappa value of 0.50, indicating weak agreement [29]. Disagreements between the reviewers were discussed with the involvement of the third reviewer until reaching consensus.

### Data Charting and Synthesis

The data charting process for this systematic scoping review involved the extraction of key entities from the included studies. These entities comprised metadata (authors’ names, year of publication, and country of study), sample demographic characteristics (sample size, age, and position, such as backs or forwards), and study characteristics (design, exposure type and length, recovery strategy used, fatigue measurement methods such as force plates or CK concentration, and the time points at which fatigue or performance was assessed). The process was conducted in four phases. First, during the training phase, two reviewers familiarised themselves with the source results and piloted the extraction form on a subset of studies to ensure all relevant data points were captured. In the extraction phase, one reviewer independently extracted the data from the studies. This was followed by the verification phase, in which another reviewer verified the extracted data for accuracy and completeness. Finally, during the reliability phase, both reviewers jointly reviewed the extracted data to resolve any discrepancies by including the third screener and discussing them until reaching consensus, ensuring consistency and reliability in the charted data. Data were synthesised descriptively, focusing on the occurrence of recovery strategies, match and rugby-specific exposures, positional differences, and fatigue/performance assessments. Findings were narratively reported to address the two primary and one secondary research questions.

To follow best practices in systematic and scoping reviews [30, 31], authors were contacted if (1) the full text of an article deemed eligible for inclusion could not be retrieved through institutional websites; and (2) data relevant for our analyses were missing from the article (for example, no data regarding the player positions are reported). If any of these conditions were present, further information was sought via email to the corresponding author. If no response was provided within two weeks, a follow-up email was sent to the corresponding author.

## Results

### Studies Selected

**Fig. 1:**
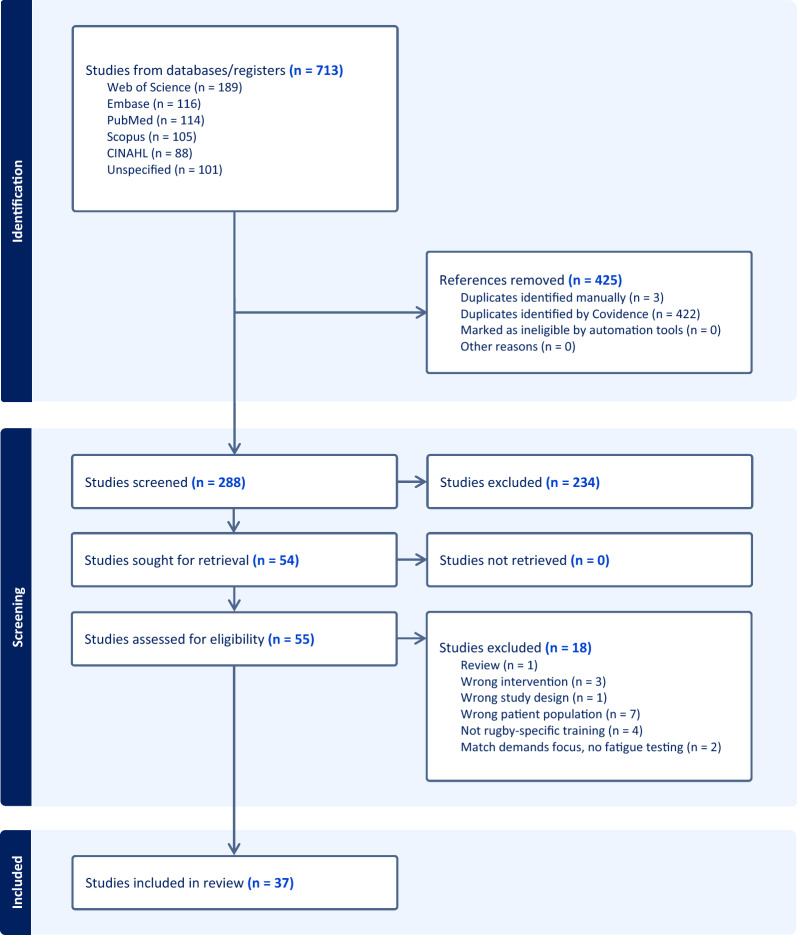
Flow chart of the studies included in the scoping review

A total of 713 articles were retrieved from the electronic searches, of which 425 were removed as duplicates (422 automatically by Covidence, and 3 manually during the full text screening). The titles and abstracts of the remaining 288 articles were independently screened and a further 234 articles were excluded from this systematic scoping review because they did not meet the eligibility criteria. The full texts of the remaining 5 articles were screened, and 36 articles were deemed suitable for inclusion in this systematic scoping review. Additionally, after contacting one author to retrieve further data, one suitable article was emailed by the author and deemed eligible for inclusion. A final number of 37 articles were included in this scoping review (Fig. 1). A total of 32 authors were contacted for further information (for example full text request or raw data), of whom 8 replied with relevant information for this review (Table 1).

**Table 1:** Characteristics of the studies included in the scoping review

Author (year), reference number and study design	Country	Sample Characteristics				Exposure		Recovery strategy	Fatigue	
		N	Age (y)	Backs	Forwards	Type	Length		Measurement	Timepoints
Aloulou et al. (2020) [44] Crossover RCT	France	19	20.7 ± 1.2	10	9	3 matches	3 weeks in season	High heat capacity mattress; whole body cryotherapy; Control	Countermovement jump; creatine kinase; delayed onset muscle soreness	Baseline and 24 h after exposure
Beavan et al. (2013) [50] Crossover RCT	New Zealand	16 (25)	25.0 ± 3.0	Not reported	Not reported	6 weeks	3 periods of 2 weeks in pre season	Electrostimulation and compression	Self report well being; testosterone, cortisol, creatine kinase	Blood samples were collected before and 36 h after matches; Questionnaires were completed three times per week
Black et al. (2018) [49] Longitudinal	New Zealand	20	22.0 ± 5.8	Not reported	Not reported	5 weeks	5 weeks in pre season	Omega-3 fatty acids to a protein-based supplement	Self report fatigue, sleep, stress and mood; Countermovement jump	Self report fatigue, sleep, stress and mood each morning of training, plus they performed countermovement jump tests once or twice per week
Crewther et al. (2019) [65] Longitudinal	Global	40 (29)	28.8 ± 2.8 (Backs); 27.8 ± 3.4 years (Forwards)	13	16	8 matches	Across 5 months in season	Natural recovery only	Salivary testosterone and cortisol concentrations; sleep duration, resting pulse rate, muscle soreness, stress, mood and motivation	Pre-match (8–9 am)
Crewther et al. (2024) [62] Longitudinal	Scotland	22	27.6 ± 3.4	10	12	3 weeks	3 weeks, in season	Natural recovery only	Mood, stress, soreness, fatigue, sleep quality	Athlete well-being was self-assessed before breakfast (served around 8–9 am) on the Tuesday, Wednesday, Friday, Saturday, and/or Sunday of each week
Cunniffe et al. (2011) [58] Pre and post	Wales	8	27.1 ± 0.8	3	5	2 matches	3 weeks, in season	Light swimming/pool work or functional rehabilitation recovery	Serum C-reactive protein, cortisol, testosterone, blood leukocytes, interleukin 6 and creatine kinase	Before and after (0, 14 and 38 h)
Daly et al. (2022) [58] Longitudinal	Ireland	11	27.1 ± 2.1	Not reported	Not reported	In season	Across 6 weeks	Natural recovery only	Direct current potential brainwave activity (via Omegawave); neuromuscular fatigue data using reactive strength index modified; profile of mood states questionnaire	Monday, Tuesday, and Thursday of each of the six weeks
Dubois et al. (2020) [45] Longitudinal	France	14	26.9 ± 1.9	8	6	35 matches	Whole season > 37 weeks	Natural recovery only	Yo-Yo intermittent recovery test, submaximal aerobic tests (50/50-test), strength tests, countermovement jump, blood sampling, and “recovery-stress” scores (RESTq)	Sequentially across all 48 weeks
Dunican et al. (2019) [38] Longitudinal	Australia	36	26 ± 3	Not reported	Not reported	1 match	1 week	Natural recovery only	Wrist-activity monitor, the ReadibandTM. Measures of alertness were calculated using the SAFTE algorithm	Every day over the week
Elloumi et al. (2003) [46] Pre and post	France	20	25.2 ± 4.2	Not reported	Not reported	1 match	6 days post-game	Natural recovery only	Salivary cortisol, salivary testosterone	6 days post-game
Grainger et al. (2020) [32] Counterbalanced sequential	England	18	25.4 ± 4.0	8	10	In season	12 weeks	Partial body cryotherapy	Self-reported well-being, muscle soreness, sleep quality and countermovement jump	Pre and 40 h post ‘real world’ training (field and gym)
Grainger et al. (2022) [8] Single group observational	England	13	27 ± 4	6	7	10 matches	10 weeks, in season	Natural recovery only	Heart rate variability indices; direct current potential; self-reported well-being	To enable comparison, data collection days were categorized in relation to their proximity to match day, ranging from match day minus 3 (MD—3), to match day plus 3 (MD + 3)
Higgins et al. (2013) [39] RCT	Australia	24	19.5 ± 0.8	Not reported	Not reported	Cyclic training and match week	6 consecutive days	Cold water immersion; Control natural recovery; Contrast baths	Countermovement jump; flexibility; circumfrance; delayed onset muscle soreness	1, 48, 72, 96 and 144 h
Higgins et al. (2011) [40] RCT	Australia	26	19 ± 1	Not reported	Not reported	In season	4 weeks	Cold water immersion; Control natural recovery; Contrast baths	300-m test, a phosphate decrement test; Subjective reports	Pre-test were conducted in the week preceeding the first game. Post-tests were conducted the week after the fourth competition game
Hills and Rogerson (2018) [33] Longitudinal	England	37	25.9 ± 4.1	17	20	In season	12 weeks	Natural recovery only	Self report well being; Countermovement jump	Compared against a baseline
Howarth et al. (2022) [54] Longitudinal	Ireland	29	24 ± 4	Not reported	Not reported	In season	32 matches (3 preseason and 29 in-season matches)	Natural recovery only	Countermovement jump	Ranging from 107–132 h post match
Hudson et al. (2020) [34] Longitudinal	England	22 (17)	25.7 ± 4.1	Not reported	Not reported	In season	7 microcycles	Natural recovery only	Indirect calorimetry was used to measure resting metabolic rate	Each morning of the competitive game week
Jones et al. (2018) [59] Longitudinal	Wales	51	22.9 ± 4.1	Not reported	Not reported	Pre season	11 week pre season	Natural recovery only	Sleep quality, energy, muscle soreness, mood, sleep hours; Countermovement jump	Twice a week (days 1 and 4) from day 4 of week 1, with one day 4 monitoring session was not conducted in week 10 (due to a change in the schedule by the team management), which gave 20 monitoring sessions in total
Kennedy and Drake (2017) [55] Longitudinal	Ireland	9	19.0 ± 1.5	Not reported	Not reported	Pre season	3 weeks	Natural recovery only	Countermovement jump	24 h and 48 h post baseline
Leduc et al. (2021) [15] Longitudinal	England	23		Not reported	Not reported	71 observations	Across a season	Natural recovery only	Sleep quality and quantity	Sleep was then measured 1 and 2 days after each match day (MD + 1 and MD + 2)
Leduc et al. (2022) [35] RCT	England	10	21.0 ± 1.3	Not reported	Not reported	In season	2 weeks	Natural recovery only	Sleep quality, fatigue, muscle soreness, stress and mood; Stroop test; Heart rate recovery; HIgh speed running to allow for the calculation of running load index as a measure of locomotor efficiency; Countermovement jump and plyometric push-up	Prior to (PRE), immediately after (POST 0 h, 14 h and 36 h post training
Lindsay et al. (2015) [51] Longitudinal pre-post	New Zealand	37	24.2 ± 2.9	Game 1 (12 backs), game 2 (7 backs), game 3 (7 backs), game 4 (7 backs) and game 5 (6 backs)	Game 1 (8 forwards), game 2 (9 forwards), game 3 (7 forwards, game 4 (10 forwards) and game 5 (8 forwards)	In season	5 consecutive matches	Cold water immersion; Swimming pool session; Compression garments	Urine and saliva samples	Urine and saliva samples were collected pre-game (within 120 min), post-game (within 60 min) and 36 h post-game (± 2 h) for five home games
Lupo et al. (2021) [64] Longitudinal pre-post	Italy	9	M 21 ± 1; F 20 ± 2	6	3	In season	One week	Natural recovery only	Self report well being; Countermovement jump, Plyometric push up	Pre and post training
Morel et al. (2015) [47] Longitudinal pre-post	France	8	23 ± 1.1	Not reported	Not reported	In season	One week	Natural recovery only	Electromyography and blood lactate	Pre and post training
Nicholls et al. (2009) [36] Longitudinal	England	16	19.3 ± 0.95	Not reported	Not reported	In season	28 days	Natural recovery only	Daily Analysis of Life Demands in Athletes questionnaire; Activation Deactivation Adjective Check List	28 sequential days
Nunes et al. (2019) [63] RCT	Brazil	22	25.2 ± 3.6	11	11	In season	One week	Cold water immersion	Tumor necrosis factor alpha, interleukin-6; Squat and countermovement jump, 10- and 30-m sprint time; soreness, perceived recovery	Obtained at pre, post, 30 min, 24, 48 and 72 h post-match
Roe et al. (2016) [37] Longitudinal	England	14	19.1 ± 1.2	Not reported	Not reported	Pre season	11 weeks	Natural recovery only	Countermovement jump, lower body strength, 40 m sprint velocity	Pre and post training
Shearer et al. (2015) [60] Longitudinal pre-post	Wales	12	24.9 ± 4.35	Not reported	Not reported	In season	One week	Natural recovery only	Mood; Testosterone, Cortisol; Countermovement jump	36 h before and 12 h, 36 h, and 60 h after a competitive rugby match
Shearer et al. (2015) [61] Pre-post	Wales	28	24.4 ± 2.9	Not reported	Not reported	In season	One week	Natural recovery only	Sleep behaviours	Sleeps were coded as follows: S1 (reference night sleep), S2 (pre-game), S3 (post-game), S4 (post-game + 1), and S5 (post- game + 2)
Tavares et al. (2018) [11] RCT	New Zealand	23	/	Not reported	Not reported	Pre season	3 weeks	Cold water immersion	Self report well being; Countermovement jump; saliva cortisol and interleukin 6	Day 1 and day 4
Tavares et al. (2018) [52] Pre-post	New Zealand	19	25.9 ± 3.6	Not reported	Not reported	In season	9 days	Natural recovery only	Self report well being; Countermovement jump	Across all 9 days
Tiernan et al. (2019) [56] Longitudinal	Ireland	19	19.7 ± 1.1	Not reported	Not reported	Pre season	10 weeks	Natural recovery only	Session rating of perceived exertion; Saliva cortisol; Sleep quality and quantity	Saliva swabs bi-weekly (Monday and Friday morning). Subjective markers of recovery were collected every morning of each training day. Session Rating of Perceived Exertion (sRPE) was taken after every training session and training load was calculated (sRPE x session duration)
Tiernan et al. (2019) [57] Longitudinal	Ireland	19	19.7 ± 1.1	Not reported	Not reported	Pre season	10 weeks	Natural recovery only	Adductor squeeze; Selected subjective markers of recovery; Rate of perceived exertion	Monday, Tuesday, and Thursday of the training week
Troester & Duffield (2019) [41] Longitudinal	Australia	27	26 ± 3	Not reported	Not reported	In season	3 microcycles	Natural recovery only	Single-leg balance and landing	At the beginning of a weekly micro-cycle 36 h after a match compared to 48 h rest without any match load
Troester & Duffield (2019) [42] Longitudinal	Australia	22	26 ± 3	Not reported	Not reported	In season	24 weeks	Natural recovery only	Single-leg balance and landing	Following 36 h recovery
Troester & Duffield (2019) [43] Longitudinal	Australia	24	25.4 ± 3.7	Not reported	Not reported	In season	7 days	Natural recovery only	Single-leg balance and landing	Testing sessions were identical and occurred on two training days separated by 7 days
Vachon et al. (2023) [48] Longitudinal	France	15 (11)	19.0 ± 1.4	Not reported	Not reported	Pre season	8 weeks	Natural recovery only	Sleep behaviours	Sequentially across all 8 weeks

### Characteristics of Included Studies

Characteristics of the included studies are displayed in Table 1. The studies included in this scoping review were published between 2003 and 2025 and assessed the fatigue experienced in the hours post-match or training (hereinafter, the exposure). As per Table 1, data were collected at different data points depending on the nature and design of the study (for example, every day over a week, or baseline, immediately post-exposure and follow-up). At the time of the literature search, the sample of rugby union players in all but one study (Nunes et al. 2019) [63] selected for the review was considered to be in Tier 1 of the World Rugby national team classification system. All studies had longitudinal, pre- and post, or randomised controlled trial designs. Studies were most frequently conducted in England (22%, = 8) [8, 15, 32–37]. Six studies (16%) were conducted in Australia [38–43], five (14%) in France [44–48], New Zealand [11, 49–52], and Ireland [53–57] and four (11%) in Wales [58–61]. Scotland [62], Brazil [63], and Italy [64] all conducted one study (3%) each. A final study (3%) included data for players playing globally [65].

### Natural Recovery

Twenty-six studies included no specific recovery intervention within their research methodology [8, 11, 15, 33–38, 42, 43, 45, 47, 48, 53–57, 59–62, 64–66]. Workload-related fatigue was reported in the majority of these papers, with the markers used to assess fatigue including sleep disturbance [15, 35, 48, 59, 60, 62], self-report wellness [8, 11, 15, 35, 36, 61, 62], perceived exertion [64], countermovement jump [8, 37, 59], plyometric push-up [35], adductor squeeze [56], single leg balance [41–43], heart rate-derived measures [8, 35] and specific rugby task performance, such as scrums and mauls [47].

Dubois et al. [45] and Tiernan et al. [57] reported a negative association between workload and physiological responses (i.e. changes in testosterone and cortisol). However, Grainger et al. [8] reported that three days post-match effects were significantly greater than one day post-match in several autonomic nervous system responses, with self-report wellness recovering at a slower time course than autonomic nervous system responses. Furthermore, match fatigue was noted by Crewther et al. [65], with higher match minutes and match involvement resulting in larger physiological and psychological stress.

Leduc et al. [15] reported an increase in fatigue over the two days post-match (measured via sleep actigraphy, wellness questionnaires, and four 60 m paced runs in 12 s), while another paper by Leduc et al. [35] reported decreases in countermovement jump mean power and self-report wellness 36 h post-training. Tavares et al. [11] reported a significant increase in muscle soreness from baseline at days 2, 3, 8, and 9 for all lower body muscles, while changes in upper body muscle soreness from baseline were only significant for days 8 and 9.

The papers by Daly et al. [53], Dunican et al. [38], Howarth et al. [54], and Kennedy et al. [55] indicated use of no specific recovery intervention within their research methodology; however, their focus was primarily on reliability, sensitivity, or usability of a fatigue measure, rather than its direct association with rugby union-specific workload. While the paper by Jones et al. [59] assessed the influence of sleep and a variety of training stimuli (i.e. resistance training), its findings were more focused on injury and illness occurrence than post rugby union training or match fatigue.

### Cold Water Immersion

Cold water immersion (CWI) was the most common recovery strategy used to enhance recovery and reduce fatigue levels [39, 40, 51, 52, 63]. Four of the studies that employed CWI took place in season and assessed recovery post-match [39, 40, 51, 63], while one took place during pre-season, when no matches occurred [52]. Four studies investigated the efficacy of CWI against a control group [39, 40, 52, 63]. Higgins et al., 2013 [39], Tavares et al., 2018 [52], and Nunes et al., 2019 [63] all reported beneficial use of CWI compared to contrast baths, whereas Higgins et al., 2011 [40] reported contrast baths as more effective for recovery, with caution advised when applying CWI exposures of 5 min. Specifically, in Higgins et al.’s (2013) study [39], they reported that contrast baths were less effective than either CWI or passive recovery in attenuating the effects of leg muscle pain after a cycle of weekly activity, including a simulated game of rugby union and a week of high-intensity training. Meanwhile, Nunes et al. [63] reported that CWI positively affected inflammation markers (i.e. TNF-α) and neuromuscular fatigue (i.e. jump height, peak power output and rate of force development from counter movement jump [CMJ]), though no effects were observed in sprint speed or perceptual recovery. Of the remaining studies that used CWI, one article compared CWI against pool-based dynamic activities as recovery strategies. In this study, Lindsay et al. [51] found that there were no differences in the recovery markers (such as salivary cortisol, total neopterin, immunoglobulin A, and myoglobin) at 36 h post-game between the different recovery protocol conditions.

### Compression Garments

Two studies utilised compression garments alongside other recovery modalities. Lindsay et al. [51] utilised compression garments in 4 different varied recovery strategies immediately post-game, with differing next-day approaches. Meanwhile, Beavan et al. [50] reported substantial benefits of the combined use of compression garments and electrostimulation compared to solely compression garments (e.g. average perceptions of energy =  effect size [ES] 0.86; enthusiasm = ES 0.80).

### Partial/Whole-Body Cryotherapy

Two studies used partial (PBC) or whole-body cryotherapy (WBC) to enhance recovery, with both studies reporting no differences in the use of PBC or WBC in reducing fatigue markers, such as assessed via self-report well-being and countermovement jump [32], or sleep architecture [44].

### Other Recovery Strategies

A variety of other recovery strategies were used in the papers that were included in this scoping review. Beavan et al. [50] was the only study that used electrostimulation to enhance recovery, reporting substantial benefits of its combined use alongside compression garments (e.g. average perceptions of energy = ES 0.86; enthusiasm = ES 0.80). One study assessed a nutritional intervention to enhance recovery during a pre-season period [49], reporting moderately beneficial effects of adding fish oil (omega-3 fatty acids) to a protein-based supplement to reduce muscle soreness. Pool-based active recovery was used in one other study as a strategy to enhance recovery [58], yet this study did not report its efficacy. Finally, Aloulou et al. [44] found statistically significant differences in fatigue markers (i.e. sleep quality) between players using an innovative high-heat capacity mattress compared to use of WBC, suggesting that the mattress may have an indirect impact on fatigue levels by improving the recovery process.

### Position-Specific Recovery Strategies

Only five studies reported positional data results [8, 33, 34, 45, 65]. One study [32] reported positional data differences between backs and forwards in relation to the WBC. In this study, participants were randomised to experimental or control groups and then crossed over to explore the effect of WBC on fatigue markers, such as sleep quality, well-being, muscle soreness, and CMJ. The results of this paper revealed that WBC did not impact the well-being scores in the backs group, whereas in the forwards group, the well-being decreased by 0.6% points after the WBC treatment, compared to a 2.40% increment when the forwards were in the control group. Regarding muscle soreness, the scores decreased by 3.8% (after WBC) and 5.5% (when in the control group) for the backs group but remained unchanged (0.0%) for the forwards when they were in the WBC group, and increased by 3.3% when the forwards were in the control group. Sleep quality decreased for all the groups in all conditions (backs in the WBC arm = − 1.6%, forwards in the WBC arm = − 5.8%; forwards in the control arm = − 1.4%), except for the backs group in the control arm condition, which had an increase of 16.4%. Finally, regarding CMJ performance, this decreased by 0.1% in the backs group after the WBC treatment, and increased by 0.7% in the forwards after exposure to the same treatment, whereas it increased by 1.2% in the backs when they were in the control arm, and decreased by 4.0% when the forwards were in the control arm.

Crewther et al. [65] followed rugby players for 8 international rugby union games. Forwards consistently reported worse scores on fatigue self-reported indicators over the course of the 8 games: sleep hours, 9.0 for the backs and 8.6 for the forwards; muscle soreness, 6.6 for the backs and 7.3 for the forwards, with higher values indicating higher levels of muscle soreness; stress levels, 6.6 for the backs and 7.2 for the forwards, with higher values indicating higher stress levels, suggesting that forwards had worse values on self-reported fatigue markers. However, regarding the physiological indicators, backs reported lower levels of testosterone (backs: 150 pg/ml, forwards: 157 pg/ml) and higher levels of cortisol (backs: 4.23 ng/ml, forwards: 4.14 ng/ml) compared to forwards, indicating that backs experienced higher levels of fatigue across the eight international matches.

Dubois et al. [45] reported that, during a training week, backs experience a greater overall workload than forwards (trivial to moderate ES, *d* = 0.4, 95%CI 0.1 to 0.7, *p* < 0.01). Backs also covered significantly greater distances, especially in the high (14.4 km/h^−1^)—and very high-speed running zones (19.9 km/h^−1^); and they covered more high-metabolic power distance. However, the research team found no significant positional differences regarding the number of accelerations and heavy impacts, but did recognise the negative influence of workload upon fatigue markers such as stress.

Hills and Rogerson [33] found that the relationship between wellness and CMJ velocity over a 12-week period was significantly stronger for backs than the forwards for each of the wellness subscales, indicating that improving players’ wellness may lead to better neuromuscular performance, in particular in backs. Even though both the wellness levels and the CMJ performance declined over the 12-week period, indicating the presence of accumulating fatigue, these results show that deliberately attempting to improve wellness levels can reduce neuromuscular fatigue.

Finally, Hudson et al. [34] reported that the backs sub-group did not show any significant changes in resting metabolic rate (RMR) or respiratory exchange ratio (RER) post-match, albeit they did show a similar pattern across the week for both sub-groups. The fatigue data showcased by Hudson et al. [34] did, however, vary across the microcycles, with suggestions that contact-based activities and eccentric strength training may be more responsible for the metabolic changes.

## Discussion

The aim of this scoping review was to explore fatigue and recovery in elite rugby union players, investigate the recovery strategies used, and explore positional differences. This scoping review provides the first attempt to summarise the recovery strategies utilised in professional rugby union (Tier 1). From the relevant articles included in this study, we were able to identify a range of strategies employed by researchers and compare their benefits. Additionally, this review is the first that seeks to identify differences in method and/or response to recovery from a playing position-specific approach. Whilst it is well established that forwards and backs show clear differences in match demands, and therefore fatigue profiles post-exercise, it is relatively unknown if these positional differences influence the recovery profiles of the players.

### Recovery Strategies

This scoping review identified a large number of studies that did not use any specific recovery strategy, or rather employed natural recovery, to reduce fatigue. In the definition of recovery, it is often highlighted that recovery modalities and managing training loads are utilised to improve return to readiness and pre-match levels of physiological capacity, a return to homeostasis [67]. The hypothesis behind utilising a number of these approaches includes an increase in the frequency of training and competition, multiple sessions per day, and limited time between sessions. However, one aspect of recovery that is often overlooked is that of natural rest, whereby the time between subsequent sessions might allow sufficient recovery of the necessary physiological systems. Regarding this point, 26 of the articles included in this scoping review did not include a specific recovery modality, but the methodological design (time points of measurement) allowed us to surmise that “natural recovery” could be assessed.

A range of variables were utilised to track workload-related fatigue in the hours and days following rugby-specific exercise (gameplay or training). Results suggest a similar pattern emerged, where negative associations were seen between workload and physiological responses; indeed, higher match minutes or involvement resulted in larger measured stress [45, 57, 65]. However, in the days following exercise, increases were seen in subjective and objective [8, 15] fatigue and muscle soreness [11], recovery was slow, and performance decrements were extended for up to 36 h [35]. Muscle soreness was even found to still be greater than baseline measures 8–9 days later [11], suggesting natural recovery might not be complete in time before subsequent sessions [8, 10]. Ultimately, these data work to support previous research that calls for an individualised or periodised approach to be taken in returning athletes to baseline levels [13, 68–70]. This is especially true in rugby, where the muscles experience high levels of eccentric movement, high-intensity static exertion, and blunt force trauma [15, 22]. Indeed, these data emphasise the premise behind the current scoping review, as it seems illogical to prescribe a singular “one-size-fits-all” approach when the physiological origin of fatigue differs so comprehensively between sports—and in this case, positions in rugby. As such, the data herein would suggest that the use of recovery modalities would be required to assist in post-exercise recovery of professional rugby union players.

The results of this scoping review identified a diversity in the recovery approaches taken, with CWI, compression garments, PBC and WBC, electrostimulation, nutritional supplements (fish oil), pool-based active recovery, and innovative mattresses utilised. These results are not too dissimilar to recovery strategies reportedly utilised in rugby union by previous reviews and articles [52, 71, 72], but such recovery strategies are significantly less than those utilised in other sports [73].

One important finding of this scoping review was that CWI is frequently used as a recovery modality in rugby union research, with benefits seen in reducing markers of fatigue, such as muscle pain, inflammatory markers, and neuromuscular fatigue. CWI has long been utilised to minimise fatigue and improve post-exercise recovery [70], primarily through its ability to remove heat from the body, reducing core and tissue temperatures, and initiating alterations in blood flow [14, 74]. Our findings align with previous research that showed that CWI has been widely suggested to benefit recovery through protection against secondary tissue damage through temperature and blood flow reductions at the site of muscle damage, thus suppressing metabolic demand and inflammatory responses [75]. Further, our findings corroborate previous research that demonstrated that CWI is implicated in reductions of pain, likely via improved analgesic effects, largely thought to be via a reduction in sensory nerve conduction velocity [76]. Meta-analyses and experimental research have regularly shown CWI to be beneficial in the recovery of physical performance [77, 78] and post-exercise soreness including delayed-onset muscle soreness [79]. The results of this scoping review support these meta-analyses with benefits seen in muscle leg pain [39], and inflammation and neuromuscular fatigue [63]. Conversely, no benefits were seen for sprint speed and perceived recovery [63]. This contrasts with a recent meta-analysis that suggested CWI could be beneficial for recovery of sprint performance following resistance exercise, and positive for perceived recovery [80]. Moreover, recent evidence suggests regular CWI might assist in perceived general well-being, with reductions in subjective worry seen from the very first immersion [81], improvements in mood [82], and fewer reported sleep disturbances [81]. However, an important caveat to this point is that the type of preceding exercise might influence the benefit seen with CWI. Therefore, this might explain the differences noted following rugby union-specific exercise.

Another common strategy used in rugby union and highlighted by this scoping review is the use of compression garments. These are typically applied to the lower (or active) limbs and provide graded external pressure to the skin [83]. Mechanisms of action are thought to include improved capillary filtration [84], increased arterial blood flow [85], and improved perception of muscle soreness and pain [86]. Interestingly, the results of this scoping review showed that compression garments with added electrostimulation was superior to compression garments alone [50]. It has been said that research into post-exercise recovery has taken a binary approach [68], assessing singular responses to an individual recovery modality. As such, this approach does not allow for investigation where a framework of periodised strategies might be applied to take advantage of different mechanisms of action to potentially further the recovery response. This might explain the superior response seen by Beaven and colleagues [50]. Sequencing multiple strategies together to optimise recovery in response to the individualised stress experienced requires further investigation.

Cryotherapy, either PBC or WBC, was also a strategy used in rugby union. However, our results showed no benefit on recovery of fatigue, well-being, sleep, and performance (CMJ). This contrasts with previous work elsewhere that shows improvements in post-exercise fatigue [87], post-exercise sleep [88], sleep per se [89], and well-being [90]. Often, contrasting evidence can be explained by differences in cooling protocols used and, therefore, the magnitude of the cooling stimulus. For example, CWI is shown to have a greater impact than PBC upon the physiological mechanisms by which they are purported to work [91–94]. This comes as no surprise given that the thermal conductivity, or heat transfer coefficient, is much greater for water (0.58 k) versus air (0.024 k) [70]. However, despite water's greater ability to remove heat from the body, evidence exists showing similar recovery profiles are seen 72 h post-exercise [94]. Moreover, it could be that the driving factor for a significant impact of cryotherapy might be the fatigue profile induced by the type of exercise, be it metabolic or mechanical in nature [79]. Our findings highlight the need to investigate through more experimental work in this area for rugby union.

Finally, this scoping review identified several studies that sought to compare certain recovery modalities against other recovery modalities [39, 40, 44, 50, 51], attempting to establish a superior modality. For example, Aloulou et al. [44] compared WBC with high-heat capacity mattresses upon sleep characteristics. Conversely, in applied practice, anecdotal reports and published articles [68] suggest that multiple recovery modalities are often used sequentially, alongside each other rather than in isolation. Our searches identified this approach, with Beaven et al. [50] sequencing electrostimulation and compression modalities. However, results utilising multiple modalities at the same time, rather than comparing modalities, were limited—suggesting current research in the area needs to do more to establish the influence of multiple sequenced modalities, versus those used in isolation.

### Position-Specific Influence

The secondary aim of this scoping review was to discuss potential positional differences in relation to fatigue and recovery strategies. When separating rugby players positionally, sub-groups of forwards and backs are most used. Our results showed that backs and forwards react differently to match play, with backs not experiencing changes in metabolic measures of RER and RMR post-exercise [34]. This is not surprising considering the volume of work that highlights differing fatigue profiles between the positions [18, 20, 22, 95], with our findings providing further support and showing that backs experience greater workloads (i.e. running greater distances at high speed) than forwards across a training week [45]. This is somewhat supported by the understanding that backs also have greater cortisol concentrations compared to forwards [65], as cortisol’s response to exercise is intensity dependent, with higher exercise intensities leading to greater release [96].

Despite this, forwards consistently report greater perceived fatigue than backs, such as less sleep, higher soreness, and greater stress [65]. Hills and Rogerson [33] suggest that the differences noted might be a result of a reduced metabolic load since backs do not experience as much contact-based activity as forwards (i.e. scrums and mauls) during match play. This suggests that the fatigue experienced might be linked to the volume of contact received. Importantly, Hills and Rogerson [33] showed that as fatigue accumulates, improved wellness is linked to improved performance. Over a 12-week period, improved wellness scores in backs were associated with greater CMJ performance, simply highlighting the importance of (and need for) recovery.

## Strengths and Limitations of the Review

This scoping review has some strengths. First, this research is the first attempt to summarise such evidence in the field of positional fatigue and associated recovery strategies in elite-level rugby union (Tier 1 players). No up-to-date research exists that practitioners working in elite-level rugby union can use as a guide to help them make more informed decisions regarding positional fatigue and the implementation of specific recovery strategies, and our study provides the first bases that practitioners can follow. Second, this scoping review included both studies examining the influence of match play and/or training on fatigue markers employing methodologies without specific recovery interventions and studies conducted in applied practice typically involving practitioners implementing recovery interventions post-match or post-training to minimise fatigue and optimise readiness for subsequent training or competition. This offered a comprehensive picture of position-specific fatigue and recovery, whilst acknowledging that recommendations for applied practice should be primarily informed by studies employing actual recovery interventions in real-world rugby contexts.

Our study has also some limitations. The limited research available for analysis within this study is, however, also a weakness of this study. Of the 36 papers identified via the search string, only 10 included deliberate recovery interventions that could be used to better understand the prevalence of recovery strategies used and their efficacy in hastening the recovery time course. Added to this, from the 713 search returns, this scoping review was able to identify only 4 studies reporting position-specific data. Several studies reported the number of forwards compared to backs that were included in the sample population [8, 32, 44, 58, 63, 64], but they failed to strategically separate the data into different positions in the results section, therefore limiting practitioners’ ability to develop insights into positional fatigue and specific recovery strategy efficacy. Lastly, it is worth noting that of the 26 studies that we assumed used natural recovery only, how these studies controlled the notion of measuring "natural recovery" only is questionable. For example, one player within any of these studies could have been more diligent with his natural recovery than others and experienced more sleep or consumed better nutrition post-match, meaning their recovery time course may have been more naturally enhanced.

## Conclusions and Implications for Practice

This systematic scoping review highlights the diverse fatigue profiles experienced by elite rugby union players, influenced significantly by positional match and training activities. For example, we know that backs [44] typically experience more high-speed running and typically demonstrate higher sprint capacities [16, 17, 22, 23] than forwards. Ultimately, the differences in match and training activities will lead to differences in motor unit and muscle fibre recruitment patterns; likely recruiting fast-twitch fibres and relying on anaerobic energy provisions [16, 17, 22, 23]. It has previously been highlighted that metabolic and mechanical fatigue can be recovered in different ways [79]. For example, Leeder and colleagues [79] outlined that eccentric exercise might create a large mechanical stress, with low metabolic cost, whilst intermittent sprint exercise might create a large amount of both mechanical and metabolic stress. It might even be suggested that this recovery profile is more metabolic in nature. This fatigue profile would appear different to that of a forward who experiences more static contact, tackling and mauling [21], perhaps producing a greater amount of muscle structure damage, inflammation, and oedema; a more mechanical fatigue profile. The complexity and variability of fatigue responses observed in forwards and backs underscore the necessity for individualised and periodised recovery approaches rather than generic, one-size-fits-all strategies. Therefore, a greater understanding of how each stressor can influence subsequent performance will assist in prescribing suitable recovery, and as such, the intervention/s should be matched to the stressor.

Whilst acknowledging that recommendations for applied practice should be primarily informed by studies employing actual recovery interventions in real-world rugby contexts, the implications for practice emerging from this review are pivotal when attempting to individualise a recovery approach (at least based on broader positions, i.e. forwards vs backs) [32]. Although natural recovery was frequently observed, specific modalities—especially CWI—showed value in mitigating muscle damage and neuromuscular fatigue. Indeed, it has previously been highlighted that some recovery strategies, particularly CWI [80], might have different outcomes depending on the nature of the preceding exercise. However, the difficulty at present is that the data to appropriately compare position-specific recovery to individual (or multiple) modalities in rugby union are either limited or unavailable. Thus, this scoping review should serve as a “call to arms” to the rugby union research community to identify position-specific responses and present position-specific data in future studies, which include standardised position fatigue terminology. Future research should also further explore the effectiveness of combined recovery modalities and investigate optimal strategies that integrate positional specificity and individualised athlete needs to enhance recovery and performance consistently across the competitive season.

## Data Availability

Not applicable.
